# Treatment of Ochronotic Osteoarthropathy and the Evaluation of Selected Lower Limb Muscle Properties, Including the Patellar Tendon: A Case Report and Mini Literature Review

**DOI:** 10.3390/jcm14134413

**Published:** 2025-06-20

**Authors:** Jaromir Jarecki, Agnieszka Tomczyk-Warunek, Agnieszka Posturzyńska, Edward Warda, Marcin Waśko, Kamil Arciszewski, Ewa Tomaszewska, Siemowit Muszyński, Jarosław Bieniaś, Monika Ostapiuk, Tomasz Skrzypek, Jacek Gągała

**Affiliations:** 1 Department of Traumatology, Orthopedics and Rehabilitation, Medical University of Lublin, 20-954 Lublin, Poland; agnieszka.posturzynska@umlub.pl (A.P.); edwardwarda37@gmail.com (E.W.); jacek.gagala@umlub.pl (J.G.); 2 Department of Radiology and Imaging, The Medical Centre for Postgraduate Education, 01-813 Warsaw, Poland; marcin@wasko.md; 3 Clinical Neurology Ward, The University Clinical Hospital No. 4 in Lublin, Jaczewskiego 8, 20-090 Lublin, Poland; kamil.arciszewski@usk4.lublin.pl; 4 Department of Animal Physiology, University of Life Sciences in Lublin, 20-950 Lublin, Poland; ewa.tomaszewska@up.lublin.pl; 5 Department of Biophysics, University of Life Sciences in Lublin, 20-950 Lublin, Poland; siemowit.muszynski@up.lublin.pl; 6 Department of Materials Engineering, Faculty of Mechanical Engineering, Lublin University of Technology, Nadbystrzycka 36, 20-618 Lublin, Poland; j.bienias@pollub.pl (J.B.); m.ostapiuk@pollub.pl (M.O.); 7 Department of Biomedicine and Environmental Research, Faculty of Medicine, John Paul II Catholic University of Lublin, 20-708 Lublin, Poland; tomasz.skrzypek@kul.pl

**Keywords:** alkaptonuria, homogentisic acid, MyotonPRO, rectus femoris, vastus medialis, patellar tendon, ochronotic osteoarthropathy, total knee arthroplasty

## Abstract

**Background/Objectives:** Alkaptonuria (AKU) is a rare genetic disorder characterized by elevated levels of circulating homogentisic acid (HGA), which accumulates in connective tissues. The musculoskeletal system is particularly susceptible to HGA deposition, often resulting in severe ochronotic osteoarthropathy, especially in the hips, shoulders, knees, and spine. However, little is known about the effects of AKU on skeletal muscle tissue. The study aimed to evaluate changes in lower limb muscles associated with AKU. **Methods:** This case report describes the treatment of ochronotic osteoarthropathy in the knee of a 73-year-old male patient. Muscle properties were assessed using the MyotonPRO device. The rectus femoris, vastus medialis, and patellar tendon were examined both preoperatively and three months postoperatively. **Results:** Following total knee arthroplasty (TKA) of the right knee, the patient demonstrated significant improvement in functional outcomes. The MyotonPRO assessment revealed measurable differences in muscle properties between the operated and non-operated limbs. Postoperative measurements indicated improvements in muscle tone, elasticity, and viscoelastic parameters in the treated limb. **Conclusions:** This case report supports the effectiveness of TKA as a treatment for ochronotic osteoarthropathy. Furthermore, it is the first study to use the MyotonPRO to assess muscle and tendon properties in a patient with AKU. These findings highlight the need for further research into the muscular effects of this rare metabolic disorder.

## 1. Introduction

### 1.1. Alkaptonuria—Definition, Causes and Epidemiology

Alkaptonuria (AKU) is a rare, inherited metabolic disorder characterized by a defect in the aromatic amino acid pathway involving tyrosine and phenylalanine. This disease holds a significant place in the history of genetics, as it was the first human disorder to be identified as an autosomal recessive condition, described by Archibald Garrod in 1902. AKU is a classic example of a monogenic autosomal recessive disorder and is marked by elevated levels of homogentisic acid (HGA) in the blood, which is subsequently excreted in the urine.

**Figure 1:**
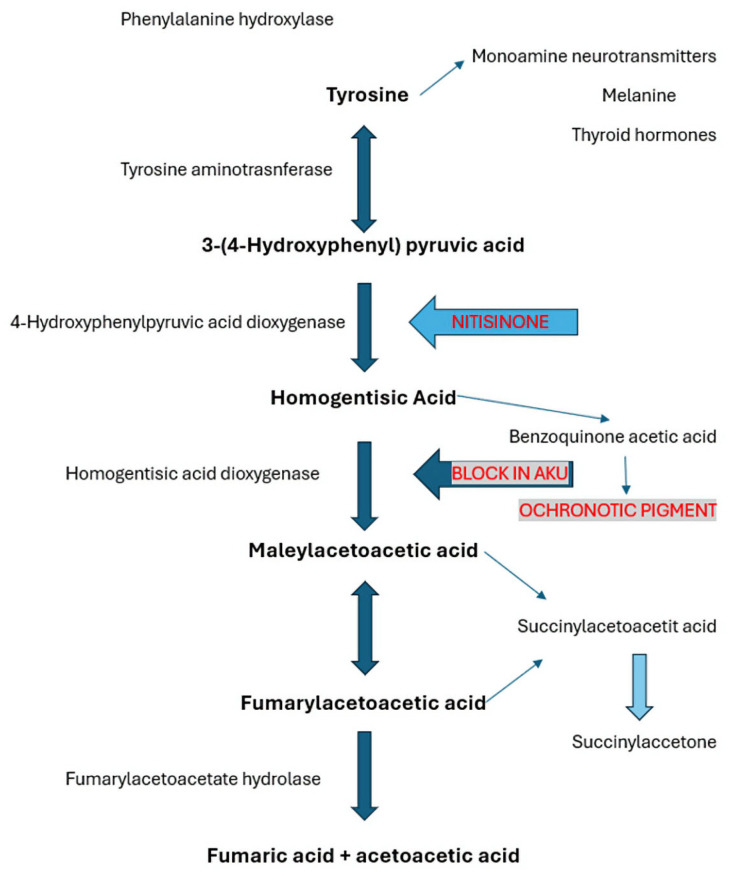
Conversion of phenylalanine to fumaric acid and acetoacetic acid. Adapted from Thapa Maheshwor et al. [2].

Mutations in the *HGD* (*homogentisate 1,2-dioxygenase*) gene encoding homogentisic acid dioxygenase (HGD) lead to a blockage in tyrosine metabolism and result in the accumulation of HGA, which in turn disrupts the function of multiple organs and accelerates tissue aging (Figure 1) [1,2]. The estimated incidence is between 1 in 250,000 and 1,000,000 live births [3]. The disease is considered endemic in Slovakia, The Czech Republic, and The Dominican Republic. Recent studies have identified a high prevalence of AKU in certain villages in Jordan and among the Romani community in India [4]. It is likely that many undiagnosed cases exist globally, particularly in regions where consanguineous marriages are common.

In most cases, signs of homogentisic aciduria can be observed from birth, manifesting as the darkening of urine that turns black upon exposure to air. This phenomenon was first documented by Scribonius in 1584, who described a young student whose urine appeared “as black as ink” [5].

Other symptoms such as blue-black pigmentation of connective tissue (ochronosis) and severe ochronotic osteoarthropathy of weight-bearing joints—including the hips, shoulders, knees, and spine—typically appear later in life, around the age of 30 [6]. In 1866, Rudolf Virchow described the autopsy of a 67-year-old man, likely deceased from congestive heart failure due to widespread atherosclerosis. In that case, intervertebral discs, the larynx, tracheal rings, menisci, articular cartilage, and atherosclerotic plaques were heavily pigmented. He coined the term “ochronosis” due to the yellowish-brown pigment seen under the microscope [5].

Pigment deposition weakens connective tissue, increasing its susceptibility to injury, degeneration, and secondary dysfunction [7]. Pigment may also deposit in the sclera (Osler’s sign), ears, skin, and heart valves—particularly the aortic valve and coronary arteries. Renal parenchyma discoloration, calculi formation, and prostatic calcifications may also be observed. Patients may suffer from more frequent bone fractures, tendon ruptures, and changes in tendon sheaths.

The severity of symptoms can vary between individuals, even among siblings, and tends to worsen with age due to ongoing HGA accumulation. Life expectancy is typically normal in patients with AKU; however, quality of life is significantly affected, mainly due to progressive joint destruction and declining mechanical joint function as a result of ochronosis [3].

### 1.2. Pathogenesis

In mammals, most dietary phenylalanine and tyrosine are metabolized by enzyme systems located primarily in the liver and kidneys. Homogentisic acid (HGA), a normal intermediate in the metabolic pathway of these two amino acids, is oxidized by homogentisate 1,2-dioxygenase (HGD) to produce maleylacetoacetic acid, which is then converted to acetoacetic acid and fumaric acid (Figure 1) [2].

A detailed analysis of the enzymatic steps involved in tyrosine metabolism in the liver of individuals with alkaptonuria has demonstrated that HGD is the only deficient enzyme. All other enzymes necessary for the conversion of tyrosine to acetoacetic acid are present and function with similar activity to those in a healthy liver [2].

The clinical manifestations and ochronotic osteoarthropathy observed in AKU are primarily attributed to the accumulation of HGA and its oxidized metabolite, benzoquinoneacetic acid. Under normal conditions, tyrosine is metabolized in the liver through multiple enzymatic steps to maleylacetoacetic acid. In ochronosis, however, a deficiency of HGD results in the buildup of HGA. This excess HGA can undergo spontaneous oxidation, or enzymatic conversion via polyphenol oxidase, to benzoquinoneacetic acid—a reactive compound that contributes to oxidative stress, free radical generation, and irreversible tissue binding.

Elevated levels of HGA and its oxidized byproducts interfere with collagen cross-linking, resulting in increased cartilage stiffness and structural weakening [2].

### 1.3. Alkaptonuria—Musculoskeletal System

The musculoskeletal system is particularly vulnerable to the deposition of HGA. It accumulates in cartilage tissue, tendons, and ligaments, impairing their function and contributing to pathological changes in bone tissue [8]. Imaging studies of joints in patients with AKU commonly reveal a narrowing of the joint space, cartilage degradation, osteophyte formation, and sclerosis of the subchondral plate [9,10].

Although the exact mechanism of HGA deposition in cartilage has not yet been fully elucidated, it is likely that it begins in the deeper layers of the articular cartilage. This process leads to a loss of elasticity and structural fragmentation, resulting in the formation of loose bodies. These, in turn, may irritate the synovial membrane, potentially leading to fibrosis. This mechanism is also thought to contribute to the development of chondromatosis, subchondral cysts, and osteophyte formation at the joint margins [8,11].

Research has shown that cartilage alterations may stem from metabolic disturbances associated with AKU, characterized by a predominance of catabolic processes. These degenerative changes typically begin in the intervertebral joints and subsequently affect larger joints [8,11,12].

In tendons and ligaments, HGA accumulation leads to structural weakening, increasing their susceptibility to rupture [9]. This process contributes to ochronotic tendinopathy, with studies estimating that 20–30% of affected individuals experience tendon or ligament tears. These changes are believed to result from HGA-induced disruption of collagen fiber cross-linking [8].

AKU also contributes to reduced bone mineral density (BMD). Studies have demonstrated that HGA intensifies bone osteolysis while osteogenesis remains unaffected, ultimately leading to bone mass loss [8]. HGA-induced microdamage to the bone matrix is especially evident in the femoral neck, where a significant decline in BMD has been observed. Conversely, lumbar spine BMD may increase, likely due to osteophyte formation and the calcification of intervertebral discs in that region [10,13].

Although the impact of AKU on muscle tissue remains unstudied, it is known that ochronotic changes within the musculoskeletal system impair physical capacity and contribute to disability [10,14]. Meanwhile, regular physical activity is crucial for maintaining healthy muscle mass [15]. Numerous studies have shown that patients with degenerative joint disease experience reduced muscle mass and quality, largely due to decreased physical activity [16,17].

One promising tool for muscle evaluation is the MyotonPRO device. It is a non-invasive, quick, and user-friendly instrument designed to assess muscle frequency (tone), biomechanical properties (stiffness and elasticity), and viscoelastic properties (relaxation and creep) [18]. The device requires only minimal training to operate. Frequency reflects intracellular muscle tension; stiffness measures the resistance of muscle tissue to deformation and contraction; elasticity reflects the muscle’s ability to absorb oscillations and return to its resting shape. Viscoelastic properties—relaxation and creep—indicate the time required for tissue deformation and recovery following contraction or external loading [18,19].

Numerous studies have confirmed that measurements obtained using MyotonPRO (Myoton AS, Talinn, Estopnia) are both reliable and reproducible [18,20,21,22,23,24]. The device has been used in patients with knee osteoarthritis (KOA), where it revealed significant changes in the biomechanical properties of muscles such as the vastus lateralis, quadriceps femoris, rectus femoris, vastus medialis, and lateral gastrocnemius [23,25,26,27,28,29]. However, no studies have yet explored the effects of HGA deposition on the biomechanical or viscoelastic properties of muscle tissue.

## 2. Case Description

The patient consented to participate in the study and to the image processing. This study was approved by the Institutional Review Board of the Bioethics Committee at the Medical University of Lublin, Lublin, Poland (approval no. KB-0024/150/11/2024).

### 2.1. Before Surgery

#### 2.1.1. Interview and Clinical Examination

A 73-year-old patient (height—170 cm, weight—80 kg, BMI—27.7 kg/cm^2^) was admitted to the hospital for chronic right knee pain caused by degenerative joint changes. The initial clinical symptoms included pain during walking, which later occurred also at rest and knee swelling, which appeared several years earlier. In order to alleviate the symptoms, the patient took nonsteroidal anti-inflammatory drugs (NSAIDs). Additionally, he underwent rehabilitation treatment: kinesitherapy, physical therapy and hydrotherapy. Despite the treatment, the disease progressed, the pain worsened, and the patient’s physical activity and walking capacity deteriorated. The patient could walk about 200 m without pain in the knee. The patient was asked to rate the pain intensity according to the Visual Analog Scale (VAS). The patient rated his pain at 8 points. Additionally, he complained of pain in the lumbar and thoracic spine. He denied pain in the hip.

**Figure 2:**
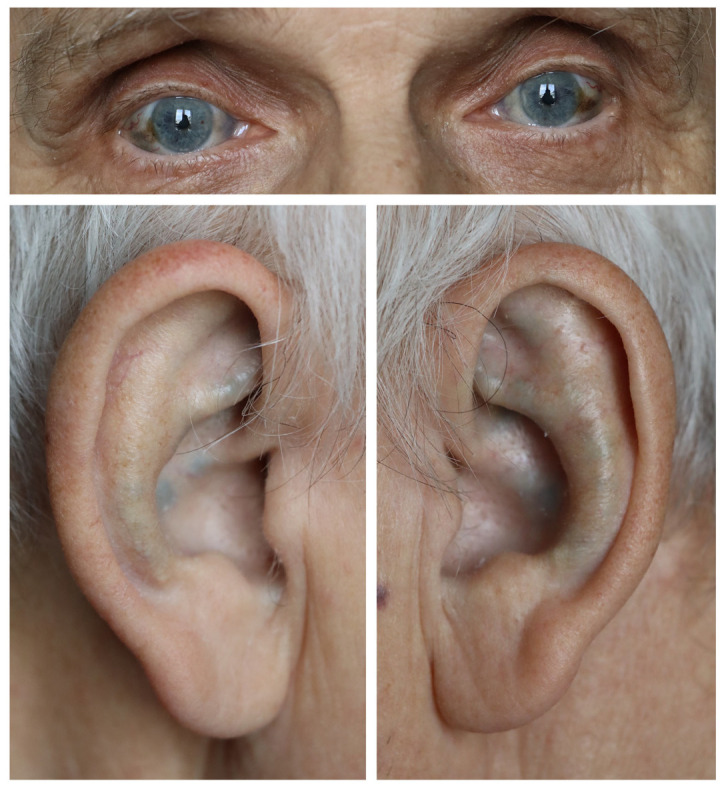
Representative photographs showing pigment deposition in the auricles and sclera.

Physical examination of the knee was performed in the supine position. The passive and active range of motion in the knee joint was 10-10-120 degrees. The knee valgus was 15 degrees. The knee joint was stable in the sagittal plane but unstable in the coronal plane. During flexion and extension movements, crepitus was clearly felt under the patella and in the lateral compartment of the knee joint. The patellar grind test was positive. Palpation of the joint space on the lateral side caused severe pain. The knee was swollen; the patella was ballotable. The Western Ontario and McMaster Universities Index of Osteoarthritis (WOMAC) score was 87. Clinical examination of the patient revealed blue-gray discoloration of the sclera of the eyes and skin lesions of the auricles, which are presented in Figure 2.

**Figure 3:**
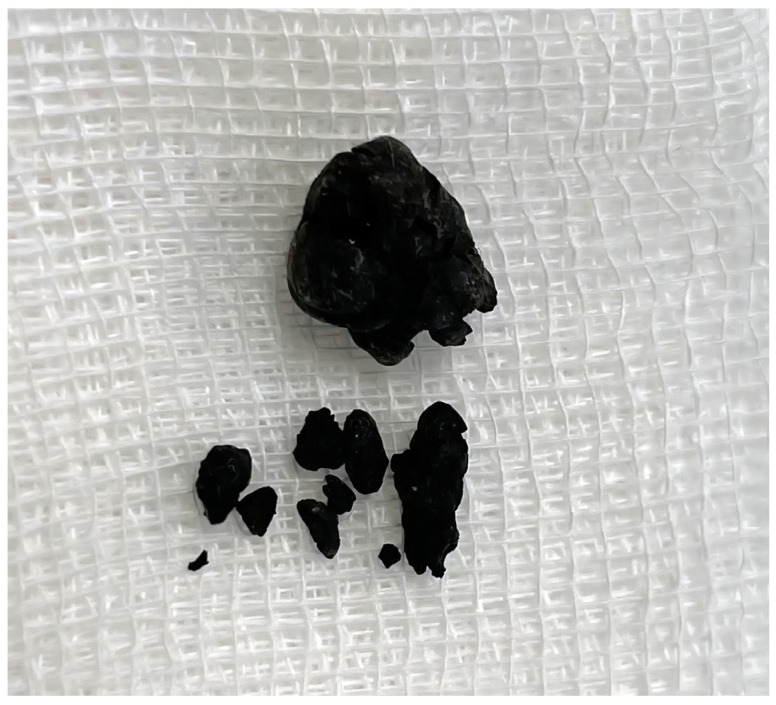
Urinary calculi.

The patient also suffers from additional conditions, including hypertension and osteoporosis. Hypertension was treated with Carvedilol at a dose of 12.5 mg per day. Osteoporosis was not treated with drugs stimulating bone formation or inhibiting bone resorption. He was taking calcium, vitamin D3 (alphadiol) and folic acid. The patient had suffered from an attack of urolithiasis several years earlier. This episode was characterized by the presence of black urinary stones (Figure 3). The patient was also assessed according to the American Society of Anesthesiologists (ASA) scale, scoring 1.

#### 2.1.2. Imaging Studies

An X-ray image of the knee joints in the anteroposterior (AP) view, taken in a standing position revealed advanced degenerative changes in both knees, characterized by a narrowing of the lateral joint spaces and osteophyte formation along the joint margins. Lateral knee X-rays showed intra-articular loose bodies and advanced degenerative changes in the patellofemoral joints. Thoracic and lumbar X-rays (AP and lateral views) demonstrated degenerative changes in the facet joints, osteophyte formation along the vertebral bodies, reduced bone density, and possible calcification of the intervertebral discs.

**Figure 4:**
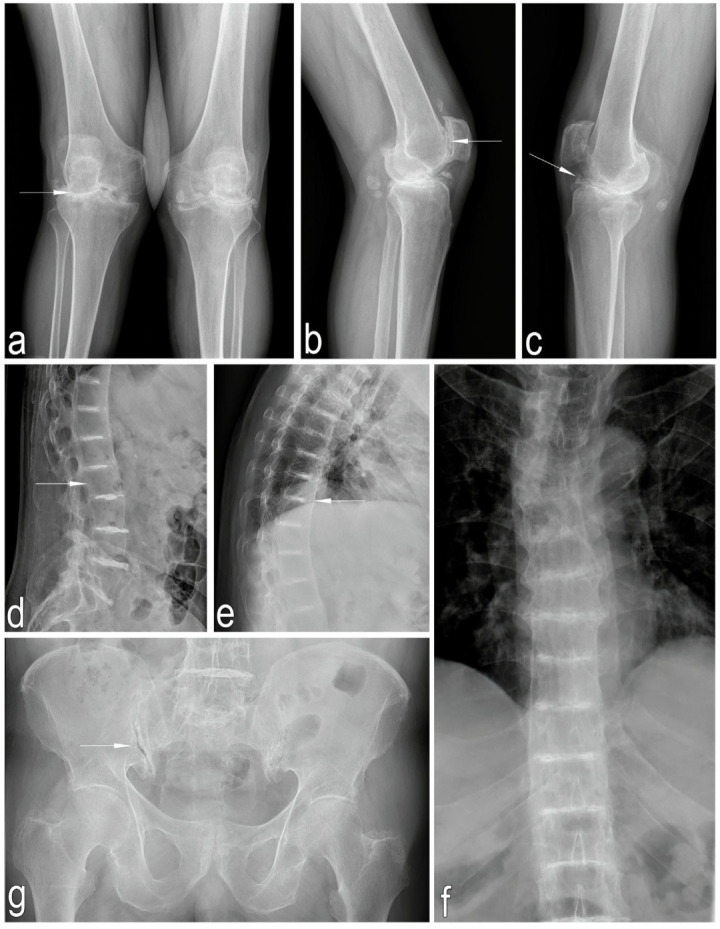
(**a**)—Anteroposterior (AP) X-rays of the knees in standing position, arrows—constriction of the articular fissure and osteophytic changes; (**b**,**c**)—Lateral views of the knees, arrows—joint free bodies and OA changes in patellofemoral joints; (**d**–**f**)—Thoracic and lumbar spine X-rays in AP and lateral views, arrows—osteoporotic bones and multilevel intervertebral disc calcification; (**g**)—AP views of the pelvis and hip joints, arrows—osteophytic changes in sacroiliac joint.

AP views of the pelvis and hip joints demonstrated early degenerative changes in the hip and sacroiliac joints, as well as reduced bone density (Figure 4).

#### 2.1.3. Laboratory Tests

**Figure 5:**
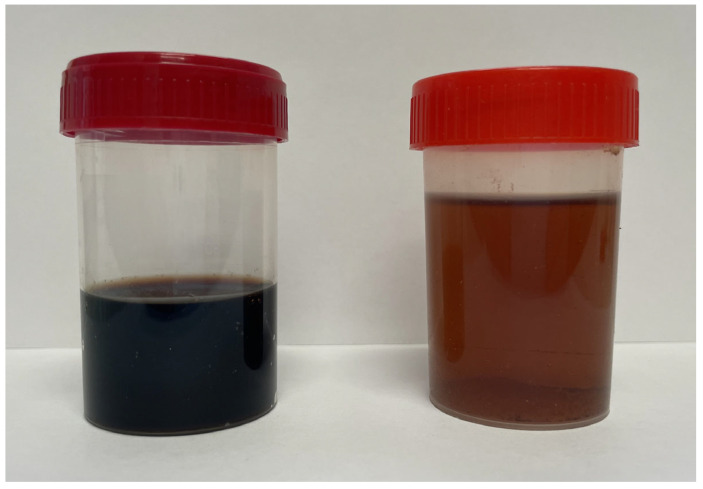
Urine samples: **left**—exposed to air; **right**—freshly collected.

Preoperative blood tests were within normal limits. The patient’s fresh urine sample did not significantly differ from that of healthy individuals. However, after 24 h at room temperature, the urine visibly darkened and turned dark brown (Figure 5). Preoperative urinalysis revealed no significant abnormalities in terms of protein, blood, or leukocyte presence.

#### 2.1.4. Densitometric Examination

The patient also underwent a bone densitometry scan. Based on the results of this examination, osteoporosis was diagnosed. The T-score for the right and left femurs was -2.3 and −2.8, respectively.

### 2.2. Surgical Treatment

The patient was placed in the supine position, and an Esmarch band was applied to the proximal third of the right thigh. Standard antiseptic preparation and draping were performed, followed by a straight 20 cm midline incision over the patella. Due to the valgus deformity of the knee, a lateral parapatellar approach was chosen.

Following joint exposure, osteophytes from the femoral condyle and hypertrophic synovial membrane were removed. Owning to the joint’s tightness and difficult access to the medial compartment, a 7 cm osteotomy of the tibial tuberosity was performed to provide full visualization of the knee joint. The cartilage tissue appeared pathologically dark—black in color. The menisci and anterior cruciate ligament (ACL) were excised, and a partial resection of the Hoffa’s fat pad was performed.

The femoral medullary canal was drilled, and an intramedullary guide rod was inserted. A resection gauge of +10 mm was applied to the distal femur, which was then resected accordingly. The tibia was anteriorly dislocated, its medullary canal was reamed, and a guide rod was inserted. Resection of the tibial plateau at the medial condyle was performed (+2 mm), after which a 9 mm trial spacer block was inserted to confirm full extension and joint stability.

Femoral and tibial preparations were completed after rotational alignment (femur-6, tibia-7, insert-9 mm). Once the Insall cage was removed, a full range of motion was confirmed. Trial implants were removed. The patella was prepared, and the femoral canal was sealed with autologous graft material.

**Figure 6:**
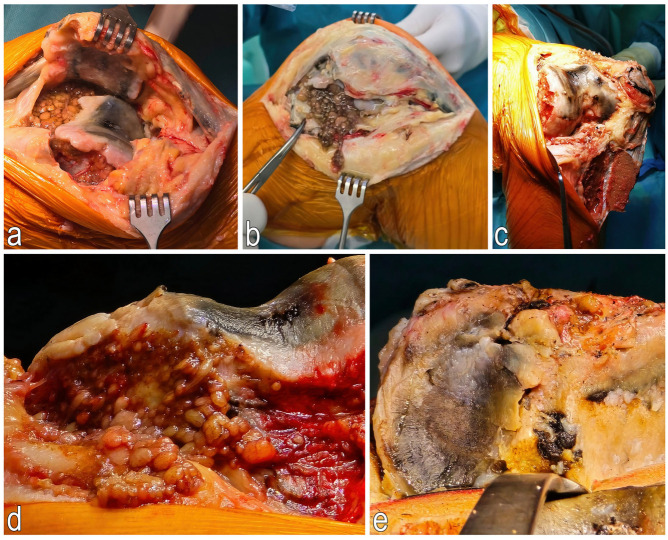
Intraoperative photographs taken during total knee arthroplasty of the right knee. (**a**)—Patellofemoral joint (anterolateral view); (**b**)—Anterolateral approach showing pathologically altered synovial membrane; (**c**)—Anterolateral view with tibial tuberosity osteotomy; (**d**)—Intercondylar fossa and synovial membrane (superior view); (**e**)—Tibial plateau (superior view), pathologically changed medial condyle.

**Figure 7:**
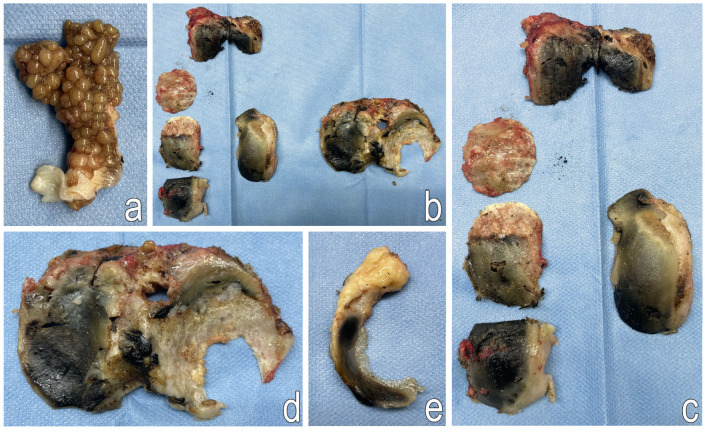
Representative images of resected, pathologically altered bone fragments from the distal femur and proximal tibia. (**a**)—Pathologically altered synovial membrane; (**b**)—Osteochondral fragments from the femur and tibia after trimming; (**c**)—Femoral osteochondral fragments; (**d**)—Trimmed tibial plateau; (**e**)—Medial meniscus.

The Triathlon Stryker PS prosthesis was implanted using Stryker bone cement in the following sizes: femur-6, tibia-7, insert-9 mm. The osteotomy site of the tibial tuberosity was stabilized with two ChM cortical screws (50 mm and 28 mm). Joint mobility was reassessed before the wound was closed in layers. After the removal of the Esmarch band, 1 g of Exacyl was administered intravenously, and a soft dressing was applied (Figure 6 and Figure 7).

#### 2.2.1. Postoperative Procedure

**Figure 8:**
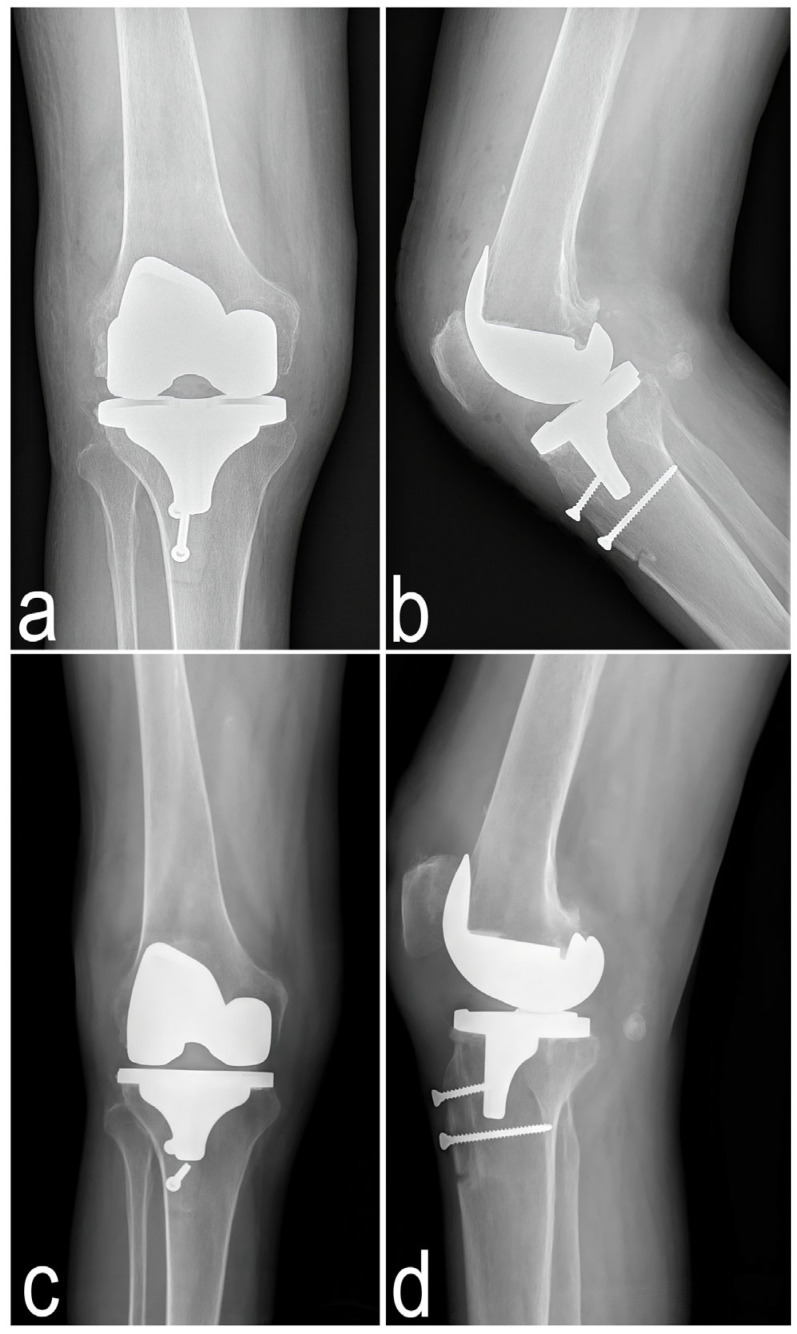
Postoperative X-ray of the right knee. The first postoperative day: (**c**)—Standing anteroposterior view; (**d**)—Lateral view. Three months after total knee arthroplasty: (**a**)—Standing anteroposterior view; (**b**)—Lateral view.

A control X-ray was taken on the first postoperative day (Figure 8c,d). Antithrombotic prophylaxis was initiated 12 h after surgery in accordance with the guidelines of the Polish Society of Orthopedics and Traumatology for the use of low-molecular-weight heparins following major joint arthroplasties. Pain was managed with NSAIDs.

Due to the tibial tuberosity osteotomy, a hinged knee brace was applied to stabilize the knee joint and protect the osteotomy site.

#### 2.2.2. Postoperative Rehabilitation

Early rehabilitation in the ward included: respiratory exercises, passive mobilization of the operated limb using a Continuous Passive Motion (CPM) (DJO Global, Lewisville, TX, USA) device, isometric exercises for the quadriceps, gluteal and trunk muscles, as well as active lower limb exercises. The patient was first mobilized on the day after surgery with a high frame walker and began walking with elbow crutches on the third postoperative day.

After hospital discharge, the patient did not attend outpatient rehabilitation. Instead, he continued performing the exercises taught during his hospital stay at home and maintained use of the passive motion device.

### 2.3. Additional Tests

During surgery, bone fragments from the distal femur and proximal tibia were collected for further analysis.

#### 2.3.1. Histological Examination

**Figure 9:**
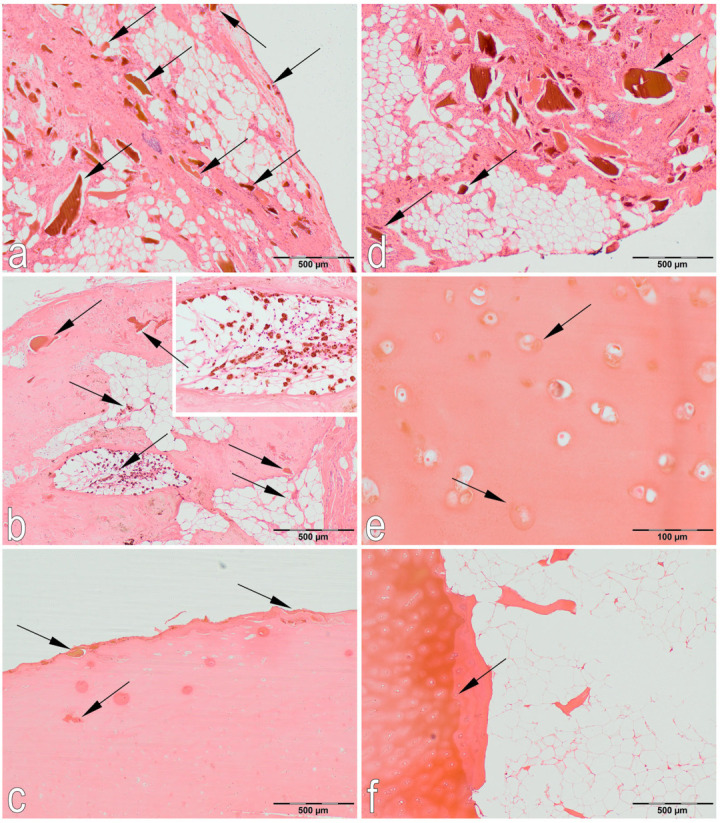
Representative H&E staining of synovium, cartilage tissue, and bone trabeculae collected from the proximal tibial epiphysis. (**a**,**d**) Synovial tissue: visible synovial steatosis and numerous sequesra of various sizes and different degrees of brown staining (arrows); (**b**) Articular cartilage: visible fatty degeneration of articular cartilage, sequesra (brown) (arrows) and foci of necrosis (arrows) present. HGA deposits visible among the fatty tissue (arrows); (**c**) Articular cartilage: the surface of the cartilage is ragged, and brown sequesra is visible in the superficial layer (arrows); (**e**) Cartilage cells in the transition zone: cartilage lacunae stained brown (arrows); (**f**) Deep zone of articular cartilage and trabecular bone: visible brown discoloration of the intercellular matrix in the deep zone of articular cartilage (arrows), few bone trabeculae, acellular bone marrow, and presence of adipose tissue.

**Figure 10:**
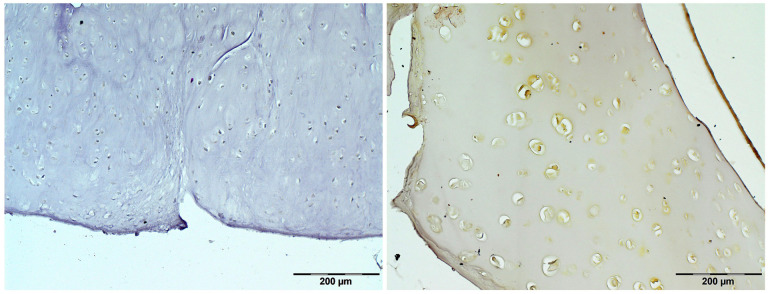
Representative immunohistochemical staining for cartilage oligomeric matrix protein (COMP). **Left**: negative control; **Right**: positive reaction in the articular cartilage matrix.

To evaluate the histopathological changes in the cartilage caused by ochronosis, hematoxylin and eosin (H&E) staining was performed (Figure 9). Histological examination confirmed the diagnosis of ochronosis due to alkaptonuria. Immunohistochemical (IHC) staining confirmed the presence of cartilage oligomeric matrix protein (COMP) in the cartilage matrix (Figure 10).

#### 2.3.2. Scanning Electron Microscopy (SEM)

**Figure 11:**
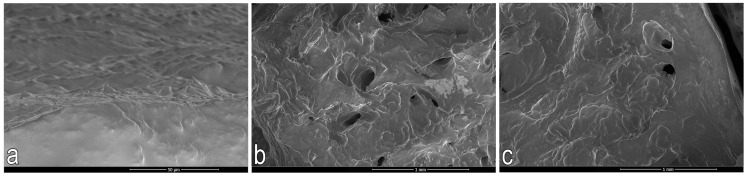
Representative SEM image of bone and cartilage tissue from the weight-bearing region of the femoral condyle. (**a**) Trabecular bone; (**b**) Articular cartilage, subchondral bone plate, and trabeculae; (**c**) Surface of the articular cartilage.

The fracture surface was analyzed using a NovaNanoSEM 450 scanning electron microscope (FEI Company, Tokyo, Japan), operating in high vacuum mode at voltages of 1.0 and 2.0 keV. The secondary electron imaging technique was used for the analysis (Figure 11).

#### 2.3.3. Nano-CT

**Figure 12:**
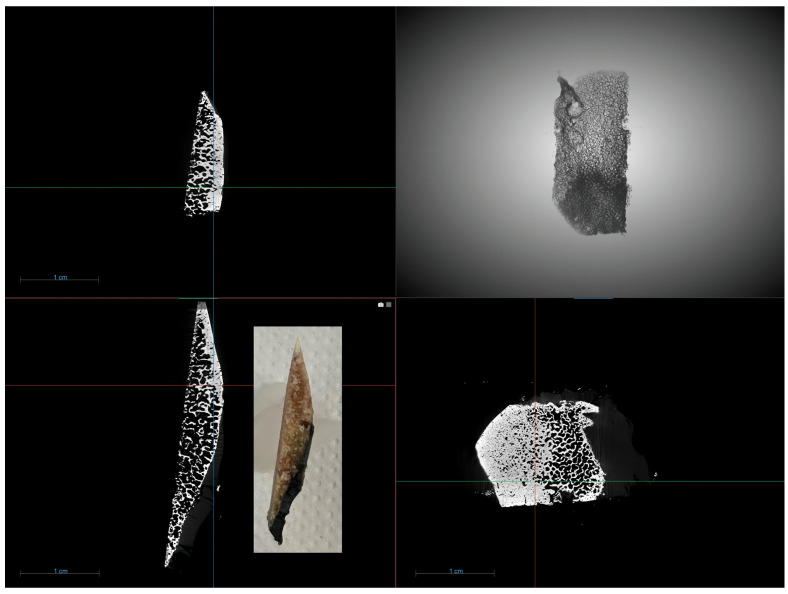
Representative nano-CT images of a selected fragment of the lateral femoral condyle from the weight-bearing region.

The internal structure was observed using an Xradia 510 Versa (Zeiss, Oberkochen, Germany) nanotomograph. The imaging parameters used for optimal visualization included a voltage of 90 kV, power of 7 W, exposure time per frame of 0.5 s, binning factor of 1, and a pixel size of 8.7 µm. No filter was applied during the scan (Figure 12).

The analysis results are presented in Table 1.

### 2.4. Follow-Up Examination 3 Months After Total Right Knee Arthroplasty

#### 2.4.1. Clinical Examination

Three months after total right knee arthroplasty, the patient was able to walk approximately 1000 m using elbow crutches without experiencing pain. He reported reduced pain and rated it at 3 out of 10 on the VAS scale.

Physical examination of the right knee was performed with the patient in the supine position. The range of motion was 5–0–120 degrees, and the knee joint was stable in all planes. The WOMAC score was 36.

Laboratory blood test results obtained during the follow-up visit were within normal limits.

#### 2.4.2. Imaging Examination

Anteroposterior and lateral X-rays of the right knee were taken three months after total knee arthroplasty (Figure 8a,b).

### 2.5. Innovative Applications of the MyotonPRO Examination for the Assessment of Muscle Condition Before and After Knee Arthroplasty in a Patient with Alkaptonuria

The MyotonPRO device is a non-invasive tool used to assess muscle tone (frequency, F), biomechanical properties (stiffness, S and elasticity, D), and viscoelastic properties (relaxation, R, and creep, C) of muscles and tendons [30].

The study was conducted by a trained and experienced examiner. Measurements were taken before surgery and three months after. Both the operated and non-operated limbs were evaluated in standing and supine positions to assess the parameters at rest and during contraction.

**Figure 13:**
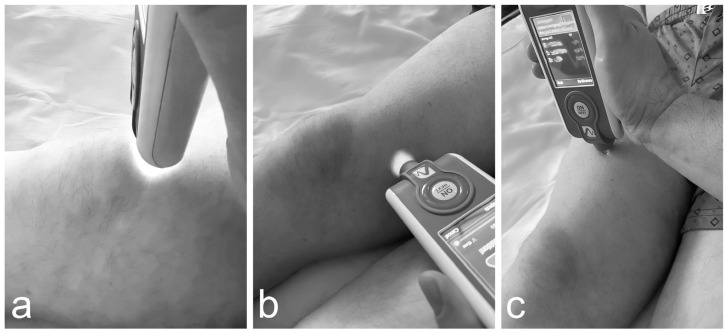
Representative photograph of the test being performed using MyotonPRO. (**a**) Patellar tendon; (**b**) Vastus medialis; (**c**) Rectus femoris.

The examined muscles included the rectus femoris and vastus medialis (quadriceps group), as well as the patellar tendon. The rectus femoris was tested 10 cm above the superior pole of the patella, the vastus medialis 5 cm medial to the joint line, and the patellar tendon at its midpoint (Figure 13).

When the probe was correctly positioned, the device generated a series of mechanical impulses (0.40 N, 10 ms) that induced soft tissue oscillations without triggering reflex muscle contractions. These oscillations were analyzed via accelerometry to calculate the biomechanical and viscoelastic properties.

#### 2.5.1.Results

##### Rectus Femoris

In the supine position before surgery, the rectus femoris on the operated side showed higher stiffness (S), elasticity (D), and frequency (F), and lower relaxation (R) and creep (C), compared to the non-operated side. Three months after TKA, the operated limb demonstrated reduced S, D, and F, and increased R and C, again in comparison with the non-operated limb (Table 2).

When comparing pre- and post-operative values for the operated limb itself, there was a reduction in F, S, and D, along with an increase in R and C (Table 2).

In the standing position, both before and three months after surgery, the operated side showed lower S, D, and F, and higher R and C compared to the non-operated side. However, comparing pre- and post-operative values for the operated side, there was no change in F, while S increased, and D, R, and C all decreased (Table 2).

##### Vastus Medialis

In the supine position before surgery, the operated limb showed lower S, D, and F, along with higher R and C compared to the non-operated side (Table 2).

Three months after right knee arthroplasty, S and F remained lower, while D, R, and C were higher than in the non-operated limb (Table 2).

When comparing pre- and postoperative values of the operated limb, the MyotonPRO results indicated increased F and S, accompanied by a decrease in D, R, and C (Table 2).

In the standing position, both before and three months after surgery, the operated limb consistently demonstrated lower S, D, and F values, and higher R and C, compared to the non-operated side (Table 2).

When comparing the pre- and post-operative results of the operated side in the standing position, there was a decrease in F and D, an increase in S, and similar values of R and C (Table 2).

##### Patellar Tendon

In the supine position, both before and three months after surgery, MyotonPRO assessment of the operated limb revealed higher F and S, and lower D, R, and C compared to the non-operated limb (Table 2).

However, when comparing the operated limb before and after surgery, a decrease in F and S and an increase in D, R, and C were observed (Table 2).

In the standing position prior to surgery, the operated limb showed lower F and S, and higher D, R, and C compared to the non-operated side (Table 2).

Three months postoperatively, the same limb demonstrated lower F, S, R, and C values, with similar D (Table 2).

In the standing position, comparison of the operated limb before and after surgery showed an increase in F and S, and a decrease in D, R, and C following TKA (Table 2).

## 3. Discussion

The case presented demonstrates that total knee arthroplasty in a patient with ochronotic arthropathy due to alkaptonuria is an effective treatment option. Functional assessment, evaluation of the joint range of motion, and pain intensity measured before and three months after surgery all indicate improvement in the patient’s condition.

It is important to note that, with the current state of medical knowledge, alkaptonuria remains an incurable disease, and only symptomatic treatment is available. Medical management focuses on alleviating various symptoms related to the deposition of HGA in tissues by inhibiting its production. To achieve this, inhibitors of 4-hydroxyphenylpyruvate dioxygenase are used. One such drug is nitisinone, which is considered a novel therapeutic agent in the treatment of alkaptonuria, as it inhibits the enzyme responsible for HGA production [7].

The deposition of homogentisic acid in the musculoskeletal system accelerates the development of degenerative changes, and symptoms may appear as early as the age of 20. In order to reduce pain and maintain proper elasticity of the soft tissues and joint mobility, physiotherapy and various manual therapy techniques are used. In advanced cases of degeneration, joint arthroplasty remains the treatment of choice [8].

In patients with alkaptonuria over the age of 40, cardiovascular and urinary system complications often develop. However, the prognosis regarding life expectancy in individuals with alkaptonuria is generally favorable. For this reason, symptomatic treatment remains essential in the management of this condition [8].

The limitations in the treatment of this disorder are associated not only with the still incomplete understanding of its etiology, but also with challenges in diagnostics. The disease is often diagnosed only when macroscopic changes appear in articular cartilage as a result of ochronotic arthropathy. Diagnosis can also be made based on the quantitative evaluation of HGA concentration in urine, as well as genetic testing confirming a mutation in the *HGD* gene. However, it should be noted that not all laboratory assessments measure urinary HGA levels, and genetic testing remains expensive. Furthermore, there is ongoing debate as to whether the results of genetic testing significantly influence the management of patients with alkaptonuria, as identifying an HGD mutation does not appear to improve treatment methods or their effectiveness.

Arthroscopy can also be used in the diagnosis of this condition. According to studies, it is an effective tool both in the diagnosis and treatment of ochronotic arthropathy. Its application may significantly alleviate joint pain and contribute to an improved range of motion in patients suffering from this condition [31,32].

As previously mentioned, the progressive degeneration of articular cartilage in alkaptonuria results from the accumulation of HGA deposits. This leads to reduced cartilage elasticity and, consequently, increased susceptibility to microtrauma. As a result, cartilage softening and fissuring may occur, penetrating its full thickness and leading to irritation and damage of the subchondral bone layer [6,12].

There are numerous reports describing cartilage damage in alkaptonuria, as well as many studies on knee joint arthropathy originating from metabolic disturbances of cartilage tissue [33]. Conversely, only a few reports mention decreased bone mineral density in patients with alkaptonuria. One study found that five out of seven patients (excluding the two youngest) were diagnosed with osteopenia [5,34,35]. That same study revealed increased levels of the receptor activator of nuclear factor kappa-B (RANK) and reduced levels of osteoprotegerin (OPG), suggesting that osteoclast activation resulting from these changes contributes to bone loss in patients with alkaptonuria [36]. Therefore, increased mechanical loading of the subchondral bone, combined with reduced cartilage elasticity, may heighten the risk of subchondral fractures due to progressive osteoporosis [6].

An innovative aspect of our case report is the use of the MyotonPRO device to assess muscle quality. As noted earlier, numerous studies have confirmed MyotonPRO as a reliable diagnostic tool for evaluating muscle tone, biomechanical, and viscoelastic properties [18,20,21,23,24,27]. To date, no studies have specifically examined how alkaptonuria affects muscle tissue, although the disease is known to cause pathological changes in cartilage, bone, ligaments, and tendons [8,11,12]. It is also known that knee osteoarthritis (KOA) significantly affects the biomechanical properties and muscle tone of the lower limbs, as evaluated using MyotonPRO [23,25,26,27,28,37]. However, similar studies have not yet been conducted in ochronotic arthropathy.

In our study, we examined two heads of the quadriceps muscle—rectus femoris and vastus medialis—as well as the patellar tendon, which are the most commonly assessed structures in existing KOA studies [23,25,26,27,28,37]. Our results showed changes in biomechanical, viscoelastic, and frequency parameters before surgery, when comparing the operated and non-operated side. In the case of the rectus femoris, in the supine position, higher stiffness, elasticity and frequency were observed with a simultaneous decrease in the relaxation and creep values in the operated limb compared to the non-operated limb. The opposite results were observed in the standing position. In the examination of the vastus medialis in contraction (standing position), lower stiffness, elasticity and frequency were observed, and increased relaxation and creep in the operated limb compared to the non-operated limb. In relaxation (standing position), the myotonPRO examination showed increased stiffness, elasticity and frequency with a simultaneous decrease in the relaxation and Creep values in the operated side.

However, our work lacks comparison of the results to the healthy population or to patients suffering from KOA. Also, as mentioned earlier, there is a lack of studies assessing lower limb muscles in patients with ochronotic arthropathy. However, a few studies have examined muscles in KOA patients with MyotonPRO. In the study by Chang et al., 2021 [23], the following quadriceps heads were examined: rectus femoris, vastus medialis, and vastus lateralis. An increase in the stiffness of the vastus lateralis was observed in patients suffering from KOA compared to the healthy group [23]. This study also showed that the stiffness of the quadriceps, especially the vastus lateralis head in patients from the KOA group, positively correlates with the degree of disability of patients (WOMAC) and with the degree of pain (VAS). However, in the case of the remaining muscles, these differences were not demonstrated [23]. The different results in our study may be due to several factors, including the small sample size (only a single patient was studied) and the absence of a healthy control. In addition, these muscles were studied in the work of Li et al., 2024 [27]. The study showed that the stiffness of the vastus lateralis was significantly higher in patients from the KOA group compared to the healthy control. When researchers in the KOA group compared the side affected with and not affected by osteoarthritis, it was observed that the frequency was significantly higher for the rectus femoris and vastus medialis in the diseased limb [27]. In our study, we also observed higher frequency when comparing the operated side with the non-operated side before TKA. In the case of the patellar tendon, there are no studies examining changes in its properties in patients suffering from AKU using MyotonPRO, although, as mentioned earlier, deposition HGA also has a negative effect on the tendon [8,9]. In the presented case report, differences were observed in the tested biomechanical and viscoelastic properties between the operated and non-operated side before and after TKA. To the best of our knowledge, this is the first study in which biomechanical and viscoelastic properties were investigated in a patient suffering from AKU after TKA. For the rectus femoris, three months postoperatively, the standing position showed similar frequency, increased stiffness, and decreased elasticity, relaxation, and creep compared to preoperative results. In the supine position, frequency, stiffness, and elasticity were reduced, while relaxation and creep increased. For the vastus medialis, in the standing position, frequency and elasticity decreased, stiffness increased, and relaxation and creep remained similar. In the supine position, frequency and stiffness increased, whereas elasticity, relaxation, and creep were reduced. In the work of Paravlić et al., 2020 [38], the changes in the rectus femoris and the vastus medialis in the results of TKA were studied using tensiomyography (TMG). It was observed that a month after, the muscle contractility was higher in the involved limb in relation to the uninvolved limb. In the case of the rectus femoris in the uninvolved side, a decrease in Dm (maximal radial displacement) was demonstrated, while the increased Tc (contraction time) was observed in the involved limb. Vastus medialis examination showed a decrease in Dm in the involved side compared to the healthy limb after TKA [37]. Both in our work and the previous Paravlić et al., 2020 [38], study, it can be observed that TKA contributes to the appearance of changes in muscle properties. The work Paravlić et al., 2020 [38] has shown that the early postoperative negative period affects the contractile properties of the muscles, but it also depends on the tested muscles. However, in our study the patient was examined three months after the operation and improvement of the examined properties of the assessed muscles was observed. The differences between our results and the previous work of Paravlić et al., 2020 [38] may result not only from the method of examination but also from the different periods in which the control examination was conducted after TKA.

Studies assessing muscle properties have shown that high intramuscular tone may restrict blood flow, leading to faster muscle fatigue and slower recovery. Greater stiffness is associated with quicker force development, and muscles with higher stiffness and elasticity are predisposed to rapid, forceful contractions. Viscoelastic properties describe the progressive elongation of muscle tissue under tensile stress [30]. Based on this, it can be concluded that TKA in a patient with ochronotic arthropathy may contribute to improved strength and regeneration of the quadriceps muscle, and increased tendon resilience during contraction, ultimately enhancing joint mobility, functional performance, and pain relief.

## 4. Conclusions

In conclusion, the case study presented highlights the importance of understanding the impact of alkaptonuria not only on the joints but also on the surrounding muscles. This is the first reported study to utilize the MyotonPRO device to assess muscle condition in a patient with this rare disorder. Our findings underscore the need for further research in this area. However, the rarity of the disease, combined with diagnostic limitations and late-stage identification, poses a significant challenge for larger-scale studies.

## Figures and Tables

**Table 1:** Results of histomorphometric analysis obtained by nano-CT of selected fragments of the lateral femoral condyle (weight-bearing region).

	Subchondral Bone Plate	Bone Trabeculae
BS/BV, 1/mm	0.700	1.15
BV/TV, %	10.0	15.4
Tb.Th, mm	7.28	4.81
Tb.Sp, mm	20.6	16.5
Tb.N, 1/mm	0.014	0.032
SMI, -	4.35	3.03
DA, -	1.67	1.46
FD, -	2.06	2.28
Conn.Dn, -	0.006	0.020
BMD, g/cm^3^	1.93	1.35

BS/BV—bone surface to bone volume ratio; BV/TV—bone volume fraction; Tb.Th—trabecular thickness; Tb.Sp—trabecular separation; Tb.N—trabecular number; SMI—structure model index; DA—degree of anisotropy; FD—fractal dimension; Conn.Dn—connectivity density; BMD—bone mineral density.

**Table 2:** Results of MyotonPRO measurements of selected muscles and the patellar tendon before and after total knee arthroplasty (TKA).

Muscle/Ligament	Muscle Condition	Side	F, Hz	S, N/m	D, -	R, ms	C, -
before TKA right knee							
Rectus femoris	Rest	Non-operated	20.5	418	0.94	13.3	0.85
		Operated	22.2	502	1.23	11.0	0.71
	Contraction	Non-operated	22.5	545	1.19	9.7	0.63
		Operated	20.6	433	1.36	12.6	0.80
Vastus medialis	Rest	Non-operated	19.3	416	1.27	13.5	0.84
		Operated	16.5	340	1.56	17.4	1.10
	Contraction	Non-operated	21.0	412	1.50	12.6	0.80
		Operated	18.9	381	1.34	13.4	0.84
Patellar tendon	Rest	Non-operated	27.2	725	0.92	7.5	0.50
		Operated	31.3	915	0.57	5.6	0.38
	Contraction	Non-operated	38.5	1094	0.35	4.3	0.31
		Operated	30.8	904	0.77	5.9	0.41
after TKA right knee							
Rectus femoris	Rest	Non-operated	21.8	454	1.12	12.0	0.77
		Operated	20.7	444	1.03	11.9	0.76
	Contraction	Non-operated	22.3	511	0.92	10.1	0.65
		Operated	20.7	448	1.01	11.7	0.74
Vastus medialis	Rest	Non-operated	19.1	445	1.36	12.5	0.80
		Operated	19.0	393	1.16	14.1	0.90
	Contraction	Non-operated	18.0	398	1.20	13.6	0.86
		Operated	18.0	398	1.20	13.6	0.86
Patellar tendon	Rest	Non-operated	25.9	716	0.95	7.7	0.51
		Operated	29.5	810	0.72	6.7	0.46
	Contraction	Non-operated	38.6	1027	0.56	5.7	0.49
		Operated	33.7	947	0.57	5.4	0.37

Legend: F—frequency; S—stiffness; D—elasticity (logarithmic decrement); R—relaxation time; C—creep.

## Data Availability

Data are contained within the article.

## Abbreviations

The following abbreviations are used in this manuscript:

AKU	Alkaptonuria
HGA	Homogentisic acid
HGO	Homogentisate 1,2-dioxygenase
VAS	Visual Analog Scale
WOMAC	Western Ontario and McMaster Universities Index of Osteoarthritis
ASA	American Society of Anesthesiologists
AP	Anteroposterior
ACL	Anterior cruciate ligament
CPM	Continuous Passive Motion
HE	Hematoxylin and eosin staining
COMP	Cartilage oligomeric matrix protein
ICH	Immunohistochemical
SEM	Scanning electron microscopy
BS/BV	Specific bone surface
BV/TV	Percent bone volume
Tb.Th	Trabecular thickness
Tb.Sp	Trabecular separation
Tb.N	Trabecular number
SMI	Structure model index
DA	Degree of anisotropy
FD	Fractal dimension
Conn.Dn	Connectivity density
BMI	Bone mineral density
F	Frequency
S	Stiffness
D	Elasticity (logarithmic decrement)
R	Relaxation time
C	Creep
RANKL	receptor activator of nuclear factor kappa-B
OPG	osteoprotegerin
KOA	Knee osteoarthritis
